# Urethane Tetramethacrylate Monomers and Nano-Fillers Enhance Dentin Bond Strength of a One-Step Universal Adhesive

**DOI:** 10.3290/j.jad.c_2432

**Published:** 2025-12-19

**Authors:** Yusuke Matsuki, Yumika Ida, Tomoki Iuchi, Wakana Oki, Kazuhide Yonekura, Yuta Utsumi, Masaomi Ikeda, Lorenzo Breschi, Keiichi Hosaka

**Affiliations:** a Yusuke Matsuki Graduate School Student, Department of Conservative Dentistry, Tokushima University Graduate School of Biomedical Sciences, Tokushima, Japan. Concept and design, wrote the manuscript, acquisition, analysis, and interpretation of data.; b Yumika Ida Assistant Professor, Department of Conservative Dentistry, Tokushima University Graduate School of Biomedical Sciences, Tokushima, Japan. Concept and design, analysis, and interpretation of data.; c Tomoki Iuchi Clinical Fellow, Department of Conservative Dentistry, Tokushima University Graduate School of Biomedical Sciences, Tokushima, Japan. Analysis and interpretation of data.; d Wakana Oki Graduate School Student, Department of Conservative Dentistry, Tokushima University Graduate School of Biomedical Sciences, Tokushima, Japan. Acquisition, analysis, and interpretation of data.; e Kazuhide Yonekura Assistant Professor, Department of Conservative Dentistry, Tokushima University Graduate School of Biomedical Sciences, Tokushima, Japan. Concept and design, acquisition, analysis, and interpretation of data.; f Yuta Utsumi Graduate School Student, Department of Conservative Dentistry, Tokushima University Graduate School of Biomedical Sciences, Tokushima, Japan. Clinical Fellow, Section of Implant and Rehabilitative Dentistry, Division of Oral Rehabilitation, Faculty of Dental Science, Kyushu University, Fukuoka, Japan. Acquisition and interpretation of data.; g Masaomi Ikeda Professor, Department of Oral Biomedical Engineering, Institute of Science Tokyo, Japan. Protocol development, wrote the manuscript, statistical analysis, and interpretation of data.; h Lorenzo Breschi Professor, Restorative Dentistry and Dental Materials at the Department of Biomedical and Neuromotor Sciences (DIBINEM), University of Bologna, Italy. Wrote the manuscript, acquisition, analysis, and interpretation of data.; i Keiichi Hosaka Professor, Department of Conservative Dentistry, Tokushima University Graduate School of Biomedical Sciences, and Division of Interdisciplinary Research for Medicine and Photonics, Institute of Post LED Photonics, Tokushima University, Japan. Affiliated Researcher, Smart Innovations for Preventive and Restorative Dentistry Research Group, Department of Operative Dentistry, Faculty of Dentistry, Chulalongkorn University, Bangkok, Thailand

**Keywords:** dentin bonding performance, multi-functional monomer, nano-filler, universal adhesive

## Abstract

**Purpose:**

This study evaluated the effects of incorporating urethane tetramethacrylate (UTMA) monomers and inorganic nano-fillers on the bonding performance and mechanical properties of a next-generation one-step universal adhesive, Clearfil Universal Bond Quick 2 (UBQ2), with a focus on clarifying its improvements over the previous formulation.

**Materials and Methods:**

Five adhesive systems were investigated: UBQ2, two experimental variants lacking either UTMA (UBQ2-U) or nano-fillers (UBQ2-F), the predecessor adhesive (Clearfil Universal Bond Quick, UBQ), and a gold-standard two-step self-etch adhesive (Clearfil SE Bond 2, SE2). Microtensile bond strength (μTBS), bond layer thickness (L), ultimate tensile strength (UTS), and water sorption (Wsp) were evaluated.

**Results:**

UBQ2 demonstrated significantly higher UTS and lower Wsp than UBQ2-U (P < 0.001 and P = 0.03, respectively), indicating enhanced mechanical resilience and hydrolytic stability due to the presence of UTMA. Compared to UBQ, UBQ2 showed improved mechanical properties while maintaining comparable μTBS. The absence of nano-fillers (UBQ2-F) significantly reduced μTBS (P < 0.05), confirming their critical role in bonding performance. Despite forming a thinner adhesive layer, UBQ2 achieved dentin bond strength comparable to SE2.

**Conclusion:**

By combining UTMA and nano-fillers, UBQ2 has improved wettability compared to conventional products, while also providing high bonding performance and mechanical properties with a one-step application. These findings support the development of universal adhesives through simplified clinical protocols and improved material properties.

**Clinical Relevance:**

This study demonstrates that incorporating nano-fillers and UTMA into a one-step universal adhesive (UBQ2) enhances mechanical strength, reduces water sorption, and maintains high dentin bond strength. Clinically, UBQ2 offers a simplified yet reliable bonding performance comparable to SE2.

Dental adhesives have evolved from complex three-step etch-and-rinse systems to simplified one-step self-etch adhesives (1-SEAs).^37^ Among these, universal adhesives (UAs) have represented a significant innovation, offering broader clinical applicability. While UAs are often classified as 1-SEAs, they are distinguished by their ability to be used in multiple bonding strategies, including self-etch, etch-and-rinse, and selective enamel etching modes, as well as their compatibility with various restorative materials.^10^

　These materials are widely used for direct restorations, indirect restorations, core build-ups, hypersensitivity treatment, and resin coating due to their excellent sealing ability and biocompatibility.^25,31^ Despite these advances, several concerns remain regarding the long-term durability of UAs, particularly those associated with their chemical composition and technique sensitivity.^10^

A key concern is the inclusion of hydrophilic monomers, which increase water sorption and may compromise the longevity of the adhesive interface.^11,16^ Clearfil Universal Bond Quick ER (UBQ) is a notable exception, as it incorporates hydrophilic amide monomers that polymerize into hydrophobic structures, thereby improving water resistance and bond durability.^21,24^ Furthermore, residual solvent can impair bonding performance,^11^ and techniques such as prolonged or warm air-blowing have been shown to enhance solvent evaporation and improve bond strength.^17,23,41^ Nevertheless, thorough air-blowing can lead to a thinner adhesive layer,^13^ which increases susceptibility to oxygen inhibition and potentially reduces polymerization and hybrid layer integrity.^26,33^

The incorporation of inorganic nano-fillers has been reported to enhance resin infiltration and increase hybrid layer thickness, thus contributing to improved bond strength.^4,9,39^ Moreover, adhesive layer formation in UAs is highly technique-sensitive, depending on solvent evaporation and adhesive viscosity, which are determined by the matrix of composite resin and filler content.^12,15,27^

Previous studies have evaluated the individual roles of nano-fillers in dentin bonding^20^ and explored multi-functional monomers with superior properties compared to conventional monomers, such as Bis-GMA and UDMA.^42^ However, the combined effects of nano-fillers and multi-functional monomers on bonding performance and mechanical properties in UAs have not been fully elucidated.

Therefore, this study aimed to evaluate the influence of adhesive composition on bonding performance and mechanical properties. Specifically, a next-generation universal adhesive, Clearfil Universal Bond Quick 2 (UBQ2), which incorporates both inorganic nano-fillers and urethane tetramethacrylate monomers (UTMA), was compared with its predecessor, UBQ, and a gold-standard 2-SEA. Additionally, two experimental adhesives were formulated by selectively removing either UTMA (UBQ2-U) or nano-fillers (UBQ2-F) from UBQ2. UTMA is a multi-functional monomer featuring a urethane backbone and four methacrylate functional groups. Its structure is designed to enhance the mechanical strength of the bonding material by increasing the crosslink density upon polymerization. The structural formula of this monomer has not been disclosed by the manufacturer. Their microtensile bond strength (μTBS), bond layer thickness (L), water sorption (Wsp), and tensile strength (UTS) were evaluated. We hypothesize that both nano-fillers and urethane tetramethacrylate monomers (UTMA) significantly contribute to the bonding performance and mechanical properties of the adhesive and that removing either component will result in a significant reduction in these properties.

## MATERIALS AND METHODS

### Adhesive Systems

Five adhesive systems were used (Table 1): Clearfil Universal Bond Quick 2 (UBQ2, Kuraray Noritake Dental, Tokyo, Japan), a commercially available one-step universal adhesive, and Clearfil Universal Bond Quick ER (UBQ, Kuraray Noritake Dental), the predecessor to UBQ2. Two experimental variants, UBQ2-U and UBQ2-F, were derived from the UBQ2 formulation and prepared in collaboration with Kuraray Noritake. In UBQ2-U, the urethane tetramethacrylate (UTMA) monomer was selectively removed. In UBQ2-F, the inorganic nano-fillers were omitted. No other components were added or substituted to compensate for the removed elements. The experimental adhesives were mixed under controlled laboratory conditions to ensure homogeneity and stored in the same type of bottle as the original UBQ2. As a control, Clearfil SE Bond 2 (SE2, Kuraray Noritake Dental) – a commercially available two-step self-etch adhesive was used. Detailed compositions and manufacturer information are provided in Table 1.

**Table 1:** The compositions of the tested adhesives and composite resin

Materials	Compositions	Manufacturer	Manufacturer’s instructions
1-UAs	Clearfil Universal Bond Quick 2(UBQ2)	10-MDP, Bis-GMA, HEMA, hydrophilic amide monomers, urethane tetramethacrylate (UTMA), colloidal silica, sodium fluoride, CQ, ethanol, water	Kuraray Noritake Dental, Japan	1. Apply bond (Waiting time: 0 s)2. Dry with mild pressure air-blowing 3. Light-cure for 10 s
UBQ2-U(UTMA Free UBQ2)	Same compositions as UBQ2 but UTMA was removed.
UBQ2-F(Filler Free UBQ2)	Same compositions as UBQ2 but filler was removed.
Clearfil Universal Bond Quick ER(UBQ)	10-MDP, Bis-GMA, HEMA, hydrophilic amide monomer, colloidal silica, ethanol, dl-camphorquinone, accelerators, water, sodium fluoride
2-SEAs	Clearfil SE Bond 2(SE2)	Primer;10-MDP, HEMA, hydrophilic aliphatic dimethacrylate, CQ, water	1. Apply primer for 20s2. Dry with mild air-blowing3. Apply bond4. Distribute evenly with mild air-blowing 5. Light-cure for 10 s
Bond; 10-MDP, Bis-GMA, HEMA, hydrophobic aliphatic dimethacrylate, CQ, initiators, colloidal silica
Composite resin	Clearfil AP-X	Bis-GMA, TEGDMA, camphorquinone, photo initiators, pigments, silanated barium glass, silanated silica	Light cure for 40s


### Adhesive Application

For UBQ2, UBQ2-U, UBQ2-F, and UBQ, the adhesive was applied to dentin and immediately air-blow dried with no waiting time, in accordance with the manufacturer’s instructions. The adhesive was then light-cured for 10 s using an LED curing unit (Pencure 2000, J. Morita; Tokyo, Japan) at 1,000 mW/cm^2^. For SE2, a self-etch primer was applied for 20 s, followed by the bonding agent and light curing for 10 s.

### Microtensile Bonding Strength (μTBS) Test

**Fig 1:**
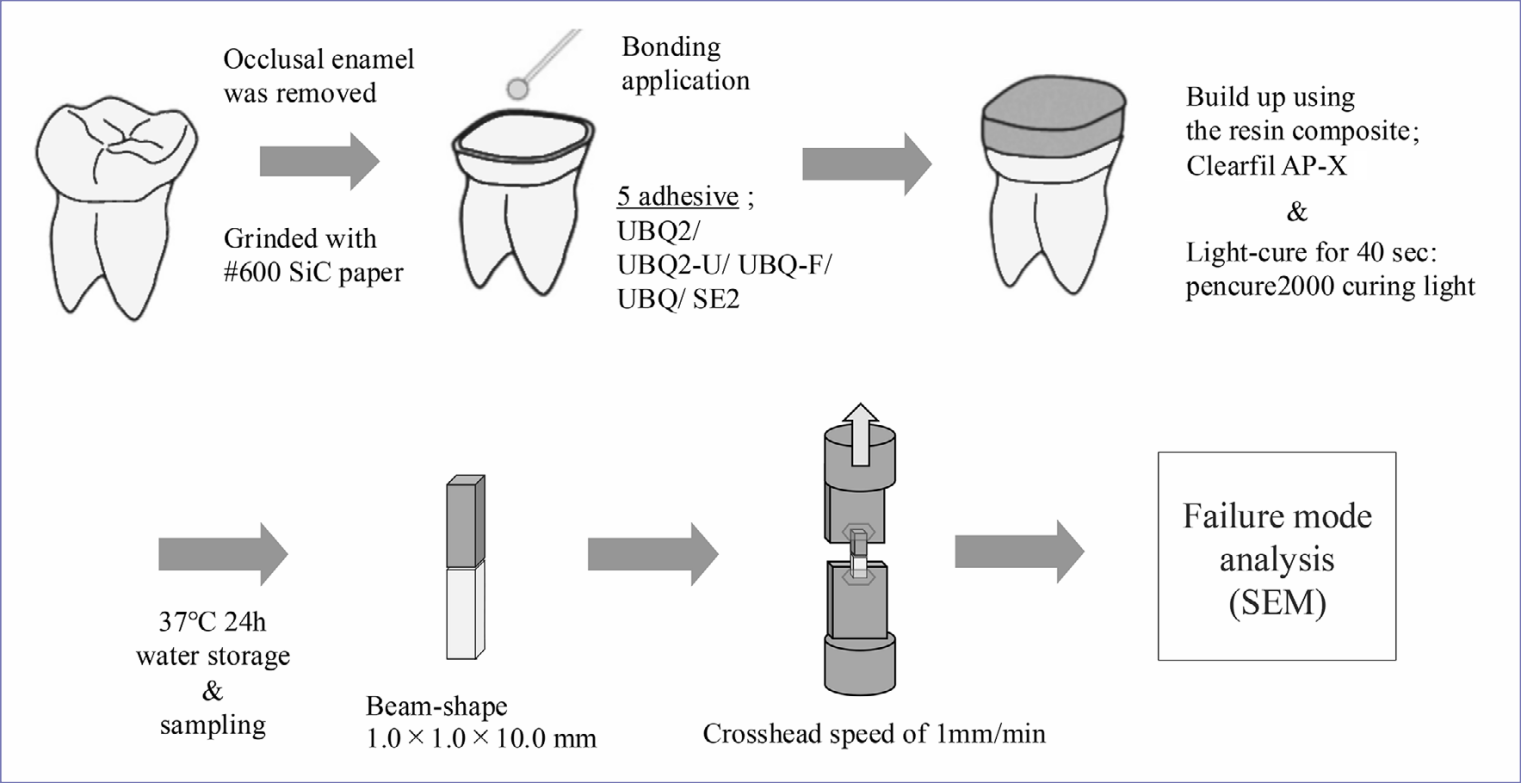
Schematic illustration of microtensile bond strength.

This study was approved by the Human Research Ethics Committee of Tokushima University (Approval No. 4503). The testing protocol followed the guidelines of the Academy of Dental Materials (ADM) for microtensile bond strength evaluation.^5^ Teeth that were too small to reliably provide sufficient specimens were excluded from this study. Twenty-five caries-free human maxillary molars collected from patients between 18 and 40 years of age were randomly assigned to one of the five experimental groups (n = 5): UBQ2, UBQ2-U, UBQ2-F, UBQ, and SE2. The teeth were stored in refrigerated water (4°C) immediately after extraction and were sectioned to expose mid-coronal dentin by removing occlusal enamel with a model trimmer under running water. The dentin surfaces were polished with 600-grit silicon carbide paper to create a standardized smear layer. Adhesives were applied and light-cured as described above. A composite resin (Clearfil AP-X; Kuraray Noritake Dental) was incrementally built up in 2-mm layers to a final height of approximately 6 mm. After storage in water at 37°C for 24 h, specimens were sectioned perpendicular to the tooth’s long axis into beams (1.0 × 1.0 mm) using a water-cooled low-speed diamond saw (Isomet, Buehler; Lake Bluff, IL, USA). From each tooth, 17 to 25 beam specimens were obtained. The dimensions of each beam were measured to two decimal places using a digital caliper (Mitutoyo, Kanagawa, Japan). Bond strength testing was performed within 1 h after beam preparation. During testing, the beams were kept on Kimwipes moistened with distilled water to prevent dehydration. Each beam was tested on a universal testing machine (EZ-SX; Shimadzu, Kyoto, Japan) at a crosshead speed of 1.0 mm/min until failure (Fig 1). Based on the reports by Loguercio et al^22^ and Armstrong et al,^5^ the average bond strength was calculated per tooth, treating each tooth as a statistical unit. Accordingly, the sample size was set to five teeth per group (n = 5). Pre-test failures, defined as specimens that fractured during trimming, mounting, or before testing, were recorded but excluded from statistical analysis.

Fracture surfaces were observed on both beams (CR side and dentin side) using a scanning electron microscope (SEM, JSM-5310, JEOL, Tokyo, Japan) at 2,000× magnification and classified as follows: CD: cohesive failure of dentin, A: adhesive failure at the resin-dentin interface, CR: cohesive failure of resin, M: mixed failure, and CA: cohesive failure within the adhesive layer. Cohesive failure within the adhesive layer was defined when more than 80% of the adhesive remained on both sides of the fracture surface. Mixed failure was characterized by a combination of interfacial failure between the adhesive resin and/or dentin, and cohesive failure within the adhesive layer.

### Bond Layer Thickness on Flat Dentin

To evaluate the adhesive layer thickness of different adhesive systems on flat dentin surfaces under microtensile bond strength (μTBS) testing conditions, the methodology followed the study by Yahagi et al,^40^ which compared the bond layer thickness between two-step and one-step self-etch adhesives (2-SEAs and 1-SEAs). Based on this method, the sample size was set at five teeth per group (n = 5).

**Fig 2:**
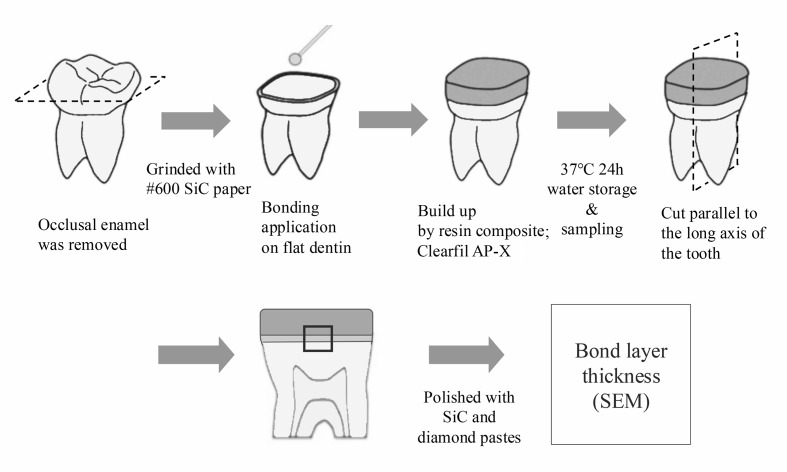
Schematic illustration of bond layer thickness verification.

An additional 25 caries-free human molars were prepared as described for the μTBS test. Following adhesive application and composite build-up, specimens were stored in water at 37°C for 24 h, then sectioned parallel to the tooth’s long axis through the center of the restoration. The cut surfaces were polished with diamond paste (DP-Paste, Struers, Ballerup, Denmark) with progressively decreasing particle sizes, ranging from 6.0 to 0.25 μm, in order to obtain specimens in which the adhesive layer between the composite and dentin could be clearly identified (Fig 2).

**Fig 3:**
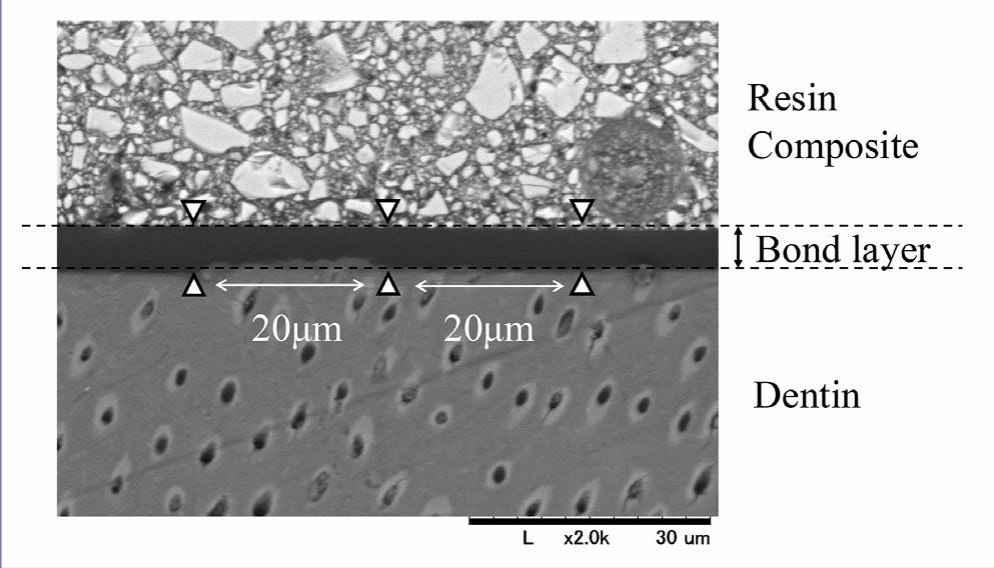
SEM analysis of adhesive layer morphology and bond layer thickness.

SEM images at 2,000× magnification were used to measure the adhesive layer thickness (L). The bond layer consists of the adhesive resin layer and the hybrid layer formed at the dentin interface. For each group (n = 5), the thinnest region was identified. Three measurements were taken: one at the thinnest point and two at positions ± 20 μm apart from that point) (Fig 3). These values were averaged to obtain the representative thickness for each specimen. The operator who performed the SEM measurements was blinded to the group assignments.

### Ultimate Tensile Strength (UTS) Test

**Fig 4:**
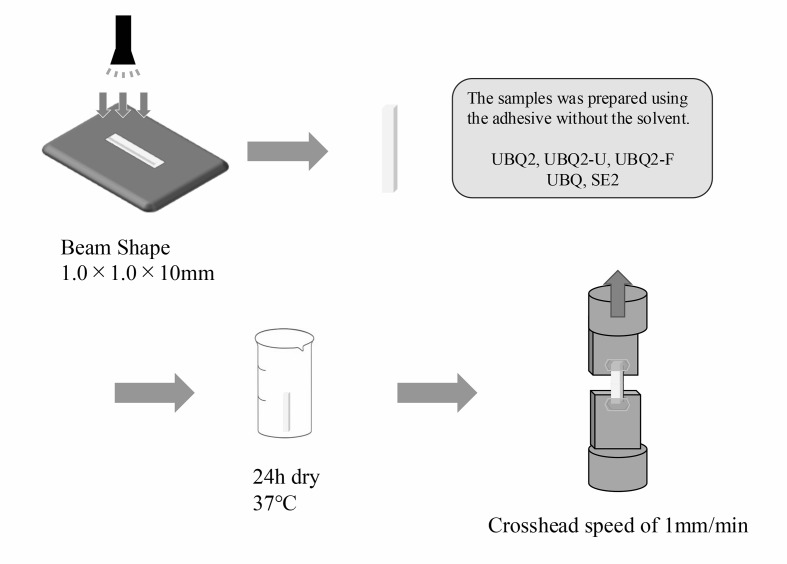
Schematic illustration of ultimate tensile strength.

Solvent-free, degassed adhesives (UBQ2, UBQ2-U UBQ2-F, UBQ, SE2) were poured into silicone molds (1.0 × 1.0 × 10.0 mm³), covered with a plastic strip, and light-cured at 1,000 mW/cm^2^ for 10 s (Pencure 2000). These solvent-free and degassed conditions were selected to isolate the intrinsic mechanical properties of the cured bonding materials and eliminate variability caused by solvent-related factors. Since SE2 does not contain solvents, this approach allowed for a direct comparison of compositional differences among the adhesives. While this setup may not fully replicate clinical conditions, it was deemed appropriate for evaluating the fundamental characteristics of the materials. Specimens were dried in a desiccator for 24 h and then tested in tension (n = 6) on EZ-SX at a crosshead speed of 1.0 mm/min (Fig 4). UTS was calculated by dividing the maximum load at failure by the cross-sectional area.

### Water Sorption Test

Adhesive materials (UBQ2, UBQ2-U, UBQ2-F, UBQ, SE2) were poured into round silicone molds (9.5 mm diameter × 2.0 mm thick), covered with a glass slide, and light-cured at 1,000 mW/cm^2^ for 20 s (Pencure 2000). To clarify the effects of compositional differences in the cured bonding materials, solvent-free and degassed adhesives were used in this test, as in the UTS experiment.

**Fig 5:**
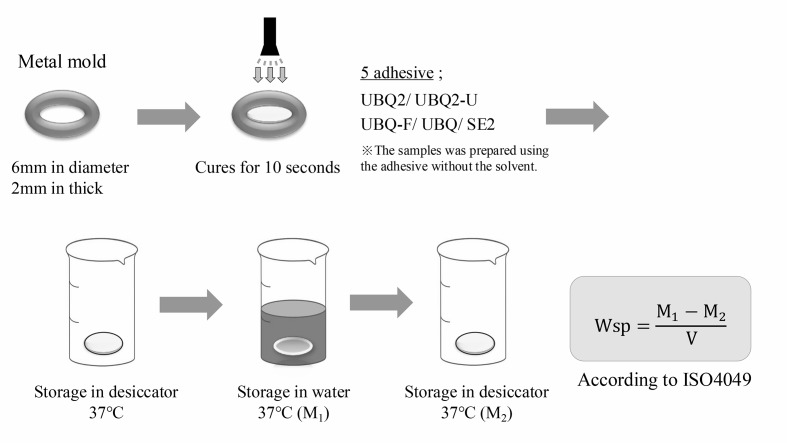
Schematic illustration of water sorption.

Immediately after curing, specimens were placed in a desiccator containing silica gel at 37°C and weighed repeatedly until a constant mass was reached. The specimen volume (V) was calculated from the measured dimensions. Then, each specimen was immersed in distilled water at 37°C for one week, removed, lightly dried, weighed (M1), and dried to a constant mass (M2) (Fig 5). Water sorption (Wsp) was calculated using the following formula:

WS = (M_1_–M_2_)/V

where M1 is the wet constant mass (μg) after water storage, M2 is the dry constant mass after desiccation, and V is the specimen volume in mm^3^.

### Statistical Analysis

The number of subjects per group (n) was calculated using a two-sided sample size determination method for each experiment. A t-test was then performed as follows:


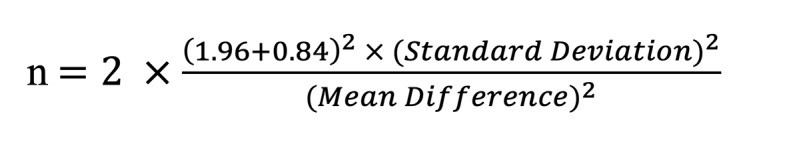


Data distribution and variance were analyzed using the Shapiro–Wilk and Levene tests. The Shapiro–Wilk test indicated that UTS and Wsp did not follow a normal distribution, while the other validation results did (P < 0.05). The Levene test showed that L-value and μTBS did not have equal variances. UTS and Wsp were statistically analyzed using Dunn’s test with Bonferroni correction at a significance level of 0.05, as a non-parametric approach. L-value and μTBS were statistically analyzed using a t-test (Welch’s method) with Bonferroni correction at a significance level of 0.05, as a parametric approach. Statistical analysis was performed using SPSS version 27.0 software (IBM, Armonk, NY, USA). The statistician conducting the analysis was blinded to the group assignments.

## RESULTS

### Microtensile Bond Strength (μTBS) Test

The results of the μTBS test, including numerical data and failure modes, are presented in Table 2. No specimen failures occurred during sample preparation for the μTBS test. According to Welch’s t-test with Bonferroni correction, UBQ2 and SE2 exhibited significantly higher bond strength than UBQ2-F (P < 0.05), while no significant differences were observed among the other groups. By contrast, the inclusion or absence of UTMA monomers had no significant effect on μTBS under these conditions. UBQ2 showed comparable bond strength to SE2 (P > 0.05).

**Table 2:** Microtensile bond strengths tested in this study

Adhesives	UBQ2	UBQ2-U	UBQ2-F	UBQ	SE2
Microtensile bond strength [MPa]	78.2^a^ (12.8)	76.8^a,b^ (2.3)	55.4^b^ (6.3)	72.7^a,b^ (18.4)	82.8^a^ (7.3)
Values are mean (SD) (N = 5). Different letters show significant differences (P < 0.05).

**Fig 6:**
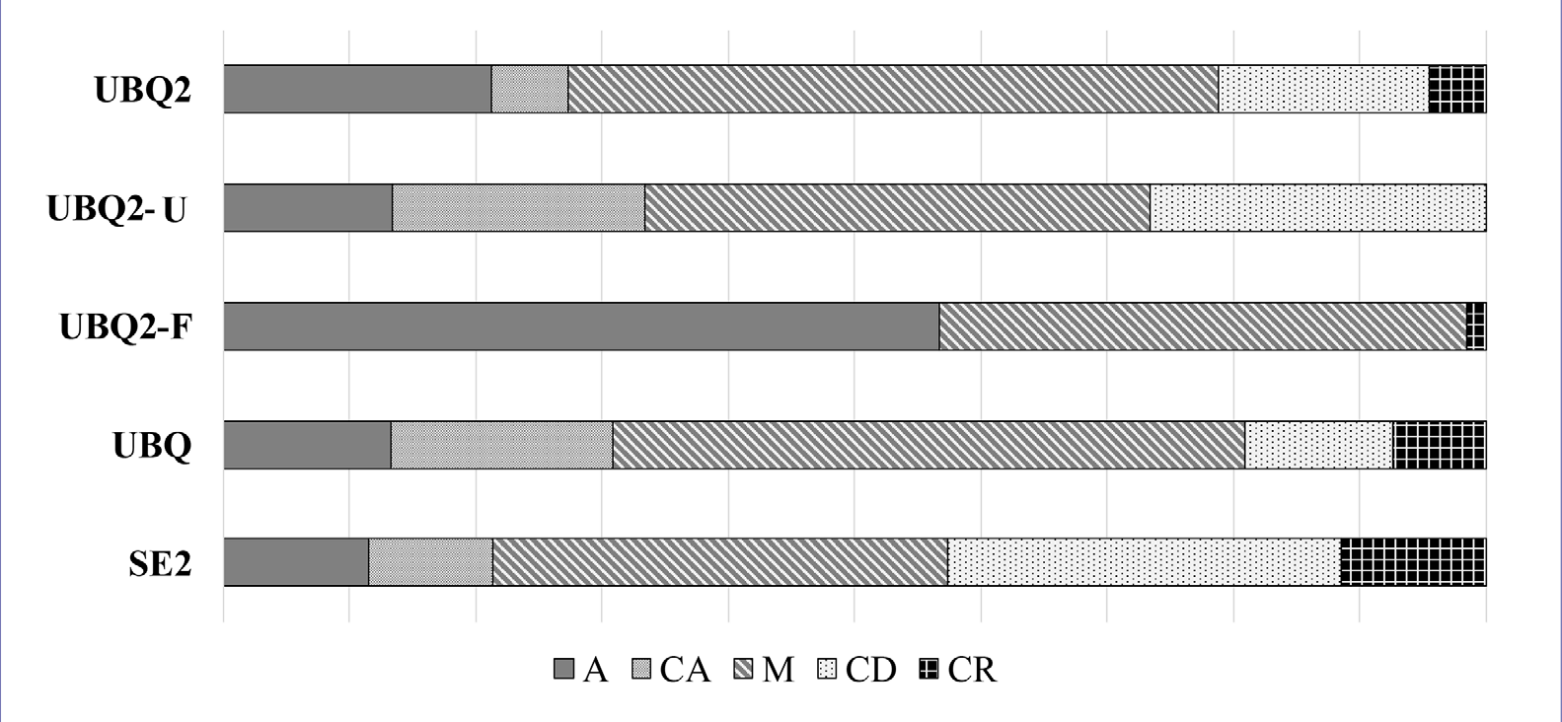
Failure mode analysis.

Failure mode analysis (Fig 6) revealed that the absence of inorganic fillers in UBQ2-F increased the proportion of interfacial failures compared with the other four groups. The relative frequency of interfacial failures followed the trend: SE2 ≤ UBQ2-U ≤ UBQ ≤ UBQ2 << UBQ2-F. Additionally, UBQ2-F exhibited neither cohesive failures in dentin (CD) nor cohesive failures in the resin (CR), whereas SE2 showed a higher frequency of cohesive failures in dentin.

### Bond Layer Thickness on Flat Dentin

**Fig 7a to e:**
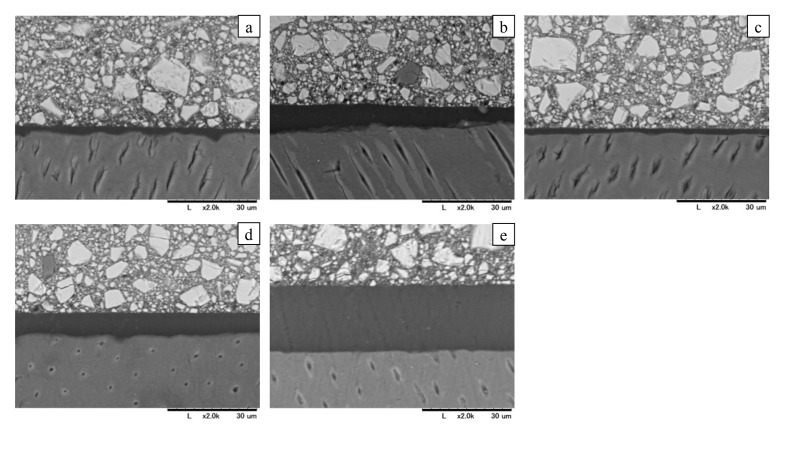
Bond layer thickness for each group. Representative SEM images (2,000× magnification) of bond layers treated with each bonding material: (a) UBQ2 (Clearfil Universal Bond Quick2); (b) UBQ2-U (UBQ2 UTMA Free); (c) UBQ2-F (UBQ2 Filler Free); (d) UBQ (Clearfil Universal Bond Quick ER); (e) SE2 (Clearfil SE Bond 2).

Representative SEM images of the adhesive interface are shown in Fig 7. The adhesive layer thickness measurements are summarized in Table 3. The statistical analysis indicates that SE2 exhibited a significantly thicker bond layer than the four 1-UAs (P < 0.01). No other significant differences were detected among all the one-step adhesives.

**Table 3:** Bond layer thickness on flat dentin

Adhesives	UBQ2	UBQ2-U	UBQ2-F	UBQ	SE2
Bond layer thickness [μm]	2.95^a^ (1.27)	6.38^a^ (1.25)	2.51^a^ (1.01)	4.64^a^ (2.37)	23.24^b^ (8.23)
Values are mean (SD) (N = 5). Different letters show significant differences (P < 0.05).

### Ultimate Tensile Strength (UTS)

Dunn’s test revealed that UBQ2 exhibited significantly higher UTS values than UBQ2-U and UBQ2-F (P < 0.01), while no significant differences were observed among the other materials (Table 4).

**Table 4:** Ultimate tensile bond strength values in this study

Adhesives	UBQ2	UBQ2-U	UBQ2-F	UBQ	SE2
UTS [MPa]	53.1^a^ (3.2)	34.9^b^ (5.3)	32.0^b^ (3.6)	43.8^a,b^ (6.1)	35.7^a,b^ (8.8)
Values are mean (SD) (N = 6). Different letters show significant differences (P < 0.05).

### Water Sorption Test

The results for water sorption (Wsp) are presented in Table 5. According to Dunn’s test, UBQ2 exhibited significantly lower Wsp than UBQ2-U (P = 0.03), whereas no significant differences were observed among the remaining groups (P > 0.05).

**Table 5:** Water sorption values in this study

Adhesives	UBQ2	UBQ2-U	UBQ2-F	UBQ	SE2
Water sorption [μg/mm^3^]	40.7^a^ (0.2)	47.7^b^ (1.5)	41.7^a,b^ (0.8)	46.4^a,b^ (0.9)	42.9^a,b^ (0.6)
Values are mean (SD) (N = 3). Different letters show significant differences (P < 0.05).

## DISCUSSION

In this study, we investigated the individual and combined effects of inorganic nano-fillers and urethane tetramethacrylate (UTMA) monomers on the performance of a one-step universal adhesive. The results showed that the absence of nano-fillers (UBQ2-F) led to a significant reduction in microtensile bond strength (μTBS) compared to UBQ2. In contrast, the removal of either nano-fillers (UBQ2-F) or UTMA (UBQ2-U) significantly decreased the mechanical properties of the adhesive. These findings support our working hypothesis that both components play essential roles: nano-fillers primarily enhance bonding performance, while UTMA contributes to mechanical reinforcement.

The incorporation of inorganic fillers into dental adhesives is widely recognized to enhance both physical and mechanical properties.^6,7,14,32,37^ Consistent with this, our results demonstrated that adhesives containing nano-fillers exhibited significantly higher microtensile bond strength (μTBS) after 24 h of water storage. This finding aligns with previous studies, which have shown that Bis-GMA/TEGDMA-based adhesives with 10-15 wt% inorganic fillers outperform unfilled formulations.^3^ However, some studies on 2-SEAs suggest that varying the filler content between 10–30 wt% does not always yield consistent benefits.^34^ The reinforcing effects of nano-fillers likely arise from multiple mechanisms: they may promote interfacial remineralization via calcium phosphate precursor formation,^29^ promoting resin tag formation,^3^ and reinforcing the adhesive layer itself.^37^ These μTBS results further support the importance of inorganic fillers in maintaining a robust resin-dentin interface. In this study, failure mode analysis revealed a higher rate of interfacial fractures in the UBQ2-F group, suggesting that the absence of nano-fillers hindered effective hybrid layer formation and compromised the integrity of the resin-dentin interface.

In our study, the removal of UTMA did not significantly reduce the μTBS, suggesting that UTMA’s contribution to immediate bond strength is limited. However, SEM analysis of flat dentin surfaces showed that UBQ2-U formulation resulted in a slightly thicker adhesive layer compared to UBQ2. This difference is likely attributable to the relatively higher viscosity of UBQ2-U, resulting from the absence of UTMA (which may have a lower viscosity than Bis-GMA). Although flat dentin surfaces were used in this study to ensure reproducibility, the lower viscosity of UBQ2 – due to the presence of UTMA – may enhance substrate wetting. This improved wettability could be particularly beneficial in clinical situations that require better adaptation to irregular geometries, such as complex cavity configurations.

Nano-fillers and multi-functional monomers demonstrated a synergistic effect in enhancing the mechanical properties of the adhesive. In this study, UBQ2 exhibited a significantly higher UTS than the variants lacking either UTMA or nano-fillers, supporting previous findings that cross-linked polymers formed from multi-functional monomers possess superior mechanical characteristics compared to those from linear monomers.^8,19,30^ The multiple reactive sites in UTMA likely contribute to a denser polymer network, while the inclusion of nano-fillers reinforces the cured matrix. The enhancement in mechanical strength is primarily attributed to the formation of strong interfacial adhesion between the nano-fillers and the surrounding resin matrix. This interfacial bonding facilitates efficient stress transfer from the matrix to the reinforcing fillers under mechanical load. As a result, crack propagation during failure is impeded, often requiring a longer and more complex path through the material, which contributes to increased fracture resistance and overall strength.^6^ Interestingly, despite expectations that lower filler content would lead to increased water uptake,^34,37^ the UBQ2-F group exhibited relatively lower water sorption. This may be attributed to the tightly cross-linked structure facilitated by UTMA, which reduces free volume for water diffusion. Furthermore, even considering the typical low filler content of the adhesive layer compared to the composite resin,^37^ the water sorption test didn’t detect any effect of filler content on Wsp, which is considered a limitation of this study.

Clearfil SE Bond2 (SE2), widely regarded as the gold standard for dentin bonding,^1,28,38^ typically exhibits higher bonding performance compared to one-step UAs.^8^ In this study, SE2 produced a significantly thicker adhesive layer than the UAs tested, consistent with previous findings that a moderately thick adhesive film can help buffer polymerization stresses.^26,33,38^ However, when the adhesive layer becomes excessively thin, particularly in one-step systems, there is a risk of incomplete polymerization at the oxygen-inhibited surface, which may compromise the integrity of the hybrid layer and reduce long-term bond durability.^8,19,30,33,38^ Therefore, we recognize that strategies such as multiple adhesive applications^34,35^ or the application of an additional hydrophobic resin layer can be effective in mitigating this issue.^2,36^

As UAs continue to be adopted across a wide range of clinical indications, achieving an optimal balance between minimal film thinness and mechanical robustness has become increasingly important.^18,37^ Our findings suggest that the combination of inorganic nano-fillers and multi-functional monomers such as UTMA enables the formation of a thin adhesive layer without compromising bond strength or mechanical performance. Based on these results, our hypothesis – that both nano-fillers and UTMA contribute substantially to bonding effectiveness and mechanical integrity and that removal of either component would degrade these properties – was partially supported. Inorganic nano-fillers were shown to significantly enhance both μTBS and UTS, while UTMA primarily improved mechanical properties without a notable effect on immediate bonding strength.

A limitation of this study is its exclusive focus on immediate bonding performance. To comprehensively evaluate the clinical reliability of these adhesives, future investigations should assess long‐term durability, including polymerization kinetics, hydrolytic degradation, and enzymatic stability. Particular attention should be given to the degree of polymerization over time and the potential formation of oxygen-inhibited or unpolymerized layers, which may compromise adhesive integrity under clinical conditions.

## CONCLUSION

A one-step universal adhesive incorporating both inorganic nano-fillers and urethane tetramethacrylate (UTMA) monomers demonstrated excellent dentin bond strength, mechanical resilience, and water resistance – performance on par with a two-step self-etch gold standard, despite forming a thinner adhesive film. These results highlight the synergistic benefits of combining nano-fillers with multi‐functional monomers and support the development of advanced universal adhesives that offer both simplified workflows and durable clinical outcomes.

### Acknowledgments

This study was supported by JST SPRING (Grant Number JPMJSP2113). Additional support was provided by the Grant-in-Aid for Scientific Research from the Ministry of Education, Culture, Sports, Science and Technology of Japan (Grant Numbers 20KK0365 and 23K09202), as well as by the Research Cluster Program of Tokushima University (Grant Numbers 2202006 and 2402003).
